# Bio-waste derived fluorescent carbon dots based biodegradable mechanically strong hydrogel for stimuli-responsive nonsteroidal anti-inflammatory drug (NSAID) delivery

**DOI:** 10.1371/journal.pone.0340974

**Published:** 2026-01-23

**Authors:** Sina M. Matalqah, Venkata Ramana Singamaneni, Patibandla Jahnavi, Umme Hani, Farhat Fatima, Rajeshwar Vodeti, Prem Shankar Gupta, Mohammad Badrud Duza, Md. Al Amin

**Affiliations:** 1 Pharmacological and Diagnostic Research Center, Faculty of Pharmacy, Al-Ahliyya Amman University, Amman, Jordan,; 2 Senior Scientist-II Department: Analytical Research and Development, Cambrex, Charles City, Iowa, United States of America; 3 Wishmen Lifesciences Pvt Ltd, Banjara Hills, Hyderabad, Khairatabad, Telangana, India,; 4 Department of Pharmaceutics, College of Pharmacy, King Khalid University (KKU), Abha, Saudi Arabia; 5 Department of Pharmaceutics, College of Pharmacy, Prince Sattam Bin Abdulaziz University, Al-kharj, Saudi Arabia; 6 Department of Pharmaceutics, School of Pharmacy, Anurag University, Hyderabad, Telangana, India; 7 Department of Pharmaceutics, Teerthanker Mahaveer College of Pharmacy, Teerthankar Mahaveer University, Moradabad, Uttar Pradesh, India; 8 Department of Pharmaceutical Chemistry, Chettinad School of Pharmaceutical Sciences, Chettinad Hospital and Research Institute, Chettinad Academy of Research and Education, Kelambakkam, Tamil Nadu, India; 9 Department of Pharmacy, Faculty of Health and Life Sciences, Daffodil International University, Dhaka, Bangladesh; Maulana Abul Kalam Azad University of Technology West Bengal, INDIA

## Abstract

In this study, biowaste derived carbon dots (BCDs) were synthesized from sugarcane bagasse via a hydrothermal route and incorporated into biodegradable, mechanically robust hydrogels for stimuli-responsive nonsteroidal anti-inflammatory drug (NSAID) delivery. BCDs, characterized by FTIR, Raman spectroscopy, and photoluminescence analyses, exhibited abundant surface functional groups and excellent fluorescence properties. The integration of BCDs into the hydrogel matrix enhanced the mechanical strength, imparted pH-responsive drug release behavior, and maintained the structural integrity under physiological conditions. In vitro release studies using ibuprofen as a model drug demonstrated faster release under acidic conditions (pH 3.8) than under neutral and alkaline pH, aligning with pathological microenvironments. The drug release kinetics were best described by the Higuchi model, indicating a diffusion-controlled mechanism. Cytotoxicity assays confirmed the high biocompatibility of both free BCDs and BCD-loaded hydrogels, with sustained cell viability over five days. The combination of bio-derived nanomaterials, tunable release properties, and excellent cytocompatibility highlight the potential of this system for targeted and environmentally sustainable drug delivery applications. This approach provides a scalable and green route for developing advanced biomedical platforms from agricultural waste with significant implications for controlled drug release and tissue-compatible therapeutic interventions.

## Introduction

Chronic inflammatory disorders, including rheumatoid arthritis, osteoarthritis, and tendinitis, represent a pervasive global health challenge that affects over 20% of the adult population worldwide and significantly contributes to long-term disability and reduced quality of life [1]. Nonsteroidal anti-inflammatory drugs (NSAIDs) such as diclofenac, ibuprofen, and ketoprofen remain first-line pharmacological agents because of their efficacy in mitigating pain, inflammation, and fever by inhibiting cyclooxygenase (COX) enzymes [2–5]. However, their systemic administration is fraught with dose-limiting adverse effects, including gastrointestinal ulcers, renal toxicity, cardiovascular risks, and hematological disturbances, which arise from non-selective biodistribution and high-dose regimens [6]. Intra-articular injections offer a promising alternative by localizing drug delivery; however, rapid synovial fluid clearance often within hours necessitates frequent reinjections, increasing infection risks, patient non-compliance, and healthcare burdens [7,8]. Consequently, the development of advanced drug delivery systems capable of sustained on-demand release at pathological sites is imperative to enhance therapeutic efficacy while minimizing off-target toxicity [9].

Hydrogels are three-dimensional (3D) hydrophilic polymer networks capable of absorbing substantial amounts of biological fluids and have emerged as quintessential platforms for controlled drug delivery, owing to their tunable porosity, biocompatibility, and structural resemblance to native extracellular matrices [10–14]. Stimuli-responsive “smart” hydrogels represent a paradigm shift, as they dynamically modulate swelling, permeability, or degradation in response to specific pathological signals [15,16]. For inflammatory disorders Endogenous triggers, such as pH fluctuations (e.g., acidic synovial microenvironments in rheumatoid joints), elevated reactive oxygen species (ROS), temperature gradients, and enzyme overexpression (e.g., matrix metalloproteinases and hyaluronidases) provide ideal release mechanisms [17,18]. Recent advances include chitosan-based thermoresponsive hydrogels for dexamethasone delivery, which exhibit phase transitions at physiological temperatures, and hyaluronic acid networks engineered for the ROS-triggered release of growth factors [19–21]. Semi-interpenetrating polymer networks (semi-IPNs), where a linear polymer interpenetrates a cross-linked network without covalent bonding, have gained traction owing to their ability to synergize the advantages of natural and synthetic polymers [22–24]. For instance, CMC-poly(N-isopropylacrylamide) (PNIPAM) semi-IPNs leverage carboxymethyl cellulose (CMC) biodegradability and PNIPAM’s temperature sensitivity of PNIPAM, whereas cellulose nanocrystal (CNC)/polyethylene glycol (PEG) systems enhance mechanical resilience and water retention [25]. Despite progress, conventional hydrogels face critical limitations, such as inadequate mechanical strength for load-bearing joints, lack of real-time monitoring capability, and reliance on non-biodegradable or synthetic components that may elicit foreign-body reactions [26].

Carbon dots (CDs), a class of zero-dimensional carbon nanoparticles (<10 nm) derived from biowaste precursors, have recently revolutionized hydrogel design by conferring multifunctionality, including intrinsic fluorescence, photoluminescence, and ROS scavenging [27]. Synthesized via sustainable routes (e.g., hydrothermal carbonization of agricultural residues), these nanomaterials offer exceptional advantages: tunable excitation/emission spectra, high quantum yields (>70% in some studies), low cytotoxicity, and surface functionalizability with carboxyl, amine, or hydroxyl groups [28–30]. When embedded in polymeric matrices, CDs mitigate aggregation-caused quenching, enhance mechanical properties through physical crosslinking, and introduce stimuli-responsive behaviors. For example, nitrogen-doped CDs (NCDs) in CMC hydrogels enable pH-dependent swelling and real-time fluorescence tracking of drug release, whereas CD-reinforced polyacrylamide networks exhibit superstretchability (842% elongation) and self-healing capabilities [31]. Recent studies have demonstrated CDs’ dual role as both optical probes and drug release modulators. Chen et al. developed oxidized arabinogel-gelatin/CD hydrogels for Cr(VI) detection, while Wen et al. created alginate-gelatin/CD films that quenched fluorescence in response to Fe(III), enabling metal ion sensing [32]. Furthermore, antioxidant properties of CDs help neutralize oxidative stress in inflamed tissues, potentially augmenting therapeutic effects of NSAIDs [33]. Biomass-derived carbon dots (B-CDs) have emerged as a sustainable and versatile class of fluorescent nanomaterials for biomedical and environmental applications. Recent reviews and experimental studies have demonstrated that a wide range of agricultural and food wastes, including fruit peels, rice husks, coconut shells, sugarcane bagasse, and other lignocellulosic residues, can be converted into carbon dots using simple, low-cost routes such as hydrothermal/solvothermal treatment, microwave-assisted synthesis, and thermal pyrolysis [34,35]. These feedstock choices strongly influence the surface chemistry of CDs (heteroatom content, oxygenated groups), size distribution, and photoluminescence, and consequently, their performance when embedded in functional matrices such as hydrogels for sensing or controlled drug release. Despite these advances, integrating biowaste derived CDs into mechanically robust, biodegradable semi-IPNs for NSAID delivery remains underexplored. Recent research has intensified the focus on semi-IPNs combining natural polysaccharides with synthetic monomers to address mechanical degradation trade-offs [36]. For instance, IA-g-poly(acrylamide)/sterculia gum semi-IPNs demonstrated negligible pH-selective swelling at gastric pH (1.2) but maximal swelling at intestinal pH (7.4), enabling targeted cetirizine delivery [37]. Similarly, CNC/PEG/PDMAA semi-IPNs loaded with gentamicin achieved sustained antibiotic release for over 72 h, accelerating wound healing via enhanced hemostasis and antimicrobial activity. However, these systems lack real-time monitoring and exhibit suboptimal mechanical strength (<100 kPa of compressive stress). To overcome this, nanocomposite reinforcement strategies have emerged: LDH incorporation into polyvinylpyrrolidone (PVP)-based semi-IPNs improves swelling stability and dye adsorption capacity, while CD-doped CMC hydrogels achieve 4.35-fold fluorescence enhancement and specific Fe(II) detection [38,39]. Despite progress, a critical gap persists in unifying five attributes: (i) biodegradability of natural polymers, (ii) mechanical resilience of dynamic joints, (iii) multi-stimuli responsiveness, (iv) real-time tracking, and (v) sustainable synthesis. No existing system seamlessly integrates biowaste derived CDs as both optical transducers and mechanical enhancers within the semi-IPN architecture for NSAID delivery [40,41].

The present study bridges this gap through a novel biodegradable semi-IPN hydrogel comprising CMC and polyacrylamide, interpenetrated with bio-waste-derived fluorescent carbon dots, for stimuli-responsive NSAID delivery. The novelty is manifested in four key aspects. First, the use of bio-waste-derived CDs introduces inherent fluorescence for non-invasive drug release monitoring, leveraging their tunable emission to correlate NSAID elution with intensity decay. Second, CDs act as multifunctional nano-crosslinkers, forming hydrogen bonds with CMC carboxyl groups and acrylamide amide moieties, augmenting compressive strength (>200 kPa) while enabling ROS/pH dual-responsiveness 416. Third, the semi-IPN architecture, where linear CMC chains interpenetrate a cross-linked polyacrylamide network, synergizes enzymatic biodegradability of CMC with the robustness of polyacrylamide, thereby addressing the fragility of polysaccharide-only gels. Fourth, the sustainable synthesis of CDs from agricultural waste (e.g., rice straw and jute fibers) aligns with circular economic principles, reducing reliance on toxic precursors. Collectively, this platform offers a paradigm shift from conventional “deliver-and-guess” systems to intelligent, trackable NSAID depots that respond to inflammatory cues, degrade harmlessly, and withstand physiological stresses that potentially revolutionize the management of chronic musculoskeletal disorders. Future studies will explore the in vivo biodistribution, long-term toxicity, and clinical translation of this multifunctional biomaterial.

## Materials and methods

### Materials

All chemicals and reagents were obtained from commercial suppliers in India and used without further purification, unless otherwise specified. Sugarcane bagasse (SCB) was collected as agricultural waste from local sugar-processing units. Carboxymethyl cellulose sodium salt (CMC; degree of substitution: 0.7–0.9, viscosity: 500–800 cP for 2% aqueous solution at 25 °C) was obtained from Bharat Starch Industries Ltd. (Hyderabad, India; Product Code: CELSOL-T). Acrylamide monomer (AAm; ≥ 98% purity, CAS 79-06-1) and N,N′-methylenebisacrylamide (MBA; > 99%, CAS 110-26-9) were purchased from Sigma-Aldrich (Germany). The initiator ammonium persulfate (APS; ≥ 98%, CAS 7727-54-0) was obtained from Sigma-Aldrich (Germany). Ibuprofen (≥99.5% purity, CAS 15687-27-1) was received as a gift from Cipla Ltd. (Mumbai, India; Batch No. IBU/AP/2203). For carbon dot synthesis, sulfuric acid (H₂SO₄; 98%, CAS 7664-93-9) was supplied by Ranbaxy Fine Chemicals Ltd. (New Delhi, India), and sodium hydroxide (NaOH; ≥ 97%, CAS 1310-73-2) was purchased from Loba Chemie Pvt. Ltd. (Mumbai, India). Dialysis was performed using cellulose ester membranes (MWCO: 3,500 Da; Himedia Laboratories, Mumbai, India). For cytotoxicity evaluation, Dulbecco’s modified Eagle’s medium (DMEM; high glucose, Gibco, USA) supplemented with 10% fetal bovine serum (FBS; Gibco, USA) and 1% penicillin-streptomycin (Thermo Fisher Scientific, USA) was used for cell culture. All aqueous solutions were prepared using deionized water (18.2 MΩ·cm resistivity) obtained from a Milli-Q Advantage A10 purification system (Merck Millipore, USA).

### Methods

#### Synthesis of bagasse derived carbon dots (BCDs).

**Fig 1:**
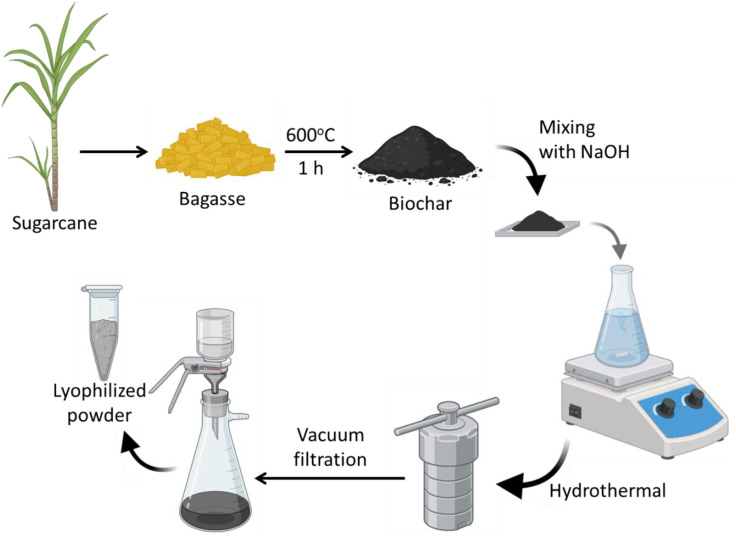
Schematic of the biowaste derived CDs synthesis by hydrothermal process.

The bagasse-derived carbon dots (BCDs) were synthesized using sugarcane bagasse as the carbon precursor (Fig 1). Initially, the bagasse was oven-dried at 60 °C for 24 h to remove residual moisture, and subsequently ground using a laboratory ball mill (Retsch PM 100, Retsch GmbH, Haan, Germany) at 300 rpm for 2 h to obtain a uniform particle size. Ten grams of the dried and milled bagasse was then pyrolyzed at 600 °C for 1 h in a muffle furnace under an inert atmosphere to produce biochar. The resulting biochar was finely ground and mixed with sodium hydroxide (NaOH) at a weight ratio of 1:4 (biochar:NaOH) to facilitate activation [42]. The activated mixture was transferred to a Teflon-lined stainless-steel autoclave containing 100 mL of deionized water and subjected to hydrothermal treatment at 200 °C for 6 h. After cooling to room temperature, the suspension was neutralized to pH 7 using dilute hydrochloric acid, followed by vacuum filtration through a 0.22 µm membrane to remove residual particulates. The filtrate was then frozen and lyophilized to yield a dry BCD powder, which was stored in airtight containers until further use. All synthesis parameters, including the precursor ratios, solvent volumes, and post-treatment steps, were precisely maintained to ensure reproducibility.

#### Synthesis of BCDs loaded nanocomposite hydrogels.

Fluorescent carbon dot (BCD)-incorporated hydrogels were fabricated through UV-initiated free-radical polymerization. First, an aqueous precursor solution was prepared by dissolving carboxymethyl cellulose (CMC, 2.0 wt%) in deionized water under magnetic stirring (500 rpm, 1 h) at 40°C. Subsequently, acrylamide monomer (20 wt%) and N,N’-methylenebisacrylamide (MBA, 0.8 mol% relative to acrylamide) were added sequentially to the CMC solution. The BCDs (0.5 wt% relative to the total polymer mass) were uniformly dispersed in the mixture via ultrasonication (30 min, 40 kHz). The photoinitiator, lithium phenyl-2,4,6-trimethylbenzoylphosphinate (LAP, 0.1%), was then dissolved in the solution under light-protected conditions. The homogeneous precursor solution was transferred into cylindrical molds (10 mm diameter × 5 mm height) and exposed to UV light (365 nm, 10 mW/cm²) for 5 min to induce cross-linking. The resulting hydrogels were immersed in deionized water for 48 h to remove unreacted components, and the water was changed every 6 h. Finally, the purified hydrogels were lyophilized (Labconco FreeZone 2.5) for 24 h and stored in desiccators until further use. For drug-loaded formulations, ibuprofen (5 wt. %) was incorporated into the precursor solution prior to UV curing.

#### Drug loading and release studies.

Ibuprofen was loaded into the hydrogels via the post-loading diffusion method. Lyophilized BCD-loaded hydrogels (100 mg) were immersed in 10 mL of ibuprofen solution (5 mg/mL in PBS, pH 7.4) and incubated at 37°C for 48 h with gentle shaking (100 rpm). The drug-loaded hydrogels were then rinsed with deionized water to remove surface-adsorbed drugs, and lyophilized. The drug loading efficiency (DLE) was calculated as 82.3 ± 3.1% using UV-Vis spectroscopy (λ = 264 nm) by measuring the residual ibuprofen concentration in the loading solution. For release studies, drug-loaded hydrogels (n = 3) were placed in 50 mL PBS (pH 7.4, 5.5) at 37°C under constant agitation (50 rpm). At predetermined intervals, 2 mL aliquots were withdrawn and replaced with fresh medium. The ibuprofen concentration was quantified spectrophotometrically (Shimadzu UV-1800) using a standard calibration curve (R² = 0.999). Cumulative release profiles were established over 8 h in different pH conditions. Data analysis was performed using the Higuchi model to determine release mechanisms. The solubility of ibuprofen in aqueous buffers of pH 1.2, 4.5, and 7.4 was determined prior to drug release experiments. Excess ibuprofen was added to each buffer and stirred at 37 °C until equilibrium was reached. The solutions were then filtered and the concentration of dissolved ibuprofen was measured using UV–Vis spectroscopy at 264 nm.

#### In vitro cytotoxicity evaluation by MTT assay.

The cytocompatibility of the BCD-loaded hydrogels was assessed using NIH/3T3 mouse fibroblast cells (ATCC CRL-1658) according to ISO 10993−5 guidelines. cells were cultured in Dulbecco’s modified Eagle’s medium (DMEM) supplemented with 10% fetal bovine serum and 1% penicillin-streptomycin at 37°C in a 5% CO₂ atmosphere. Sterilized hydrogel extracts were prepared by incubating 1 cm² samples in 5 mL culture medium for 24 h at 37°C. Fibroblasts (1 × 10⁴ cells/well) were seeded in 96-well plates and exposed to serial dilutions (100%, 50%, and 25%) of the hydrogel extracts for 24 and 48 h. After treatment, 20 μL MTT solution (5 mg/mL in PBS) was added to each well and incubated for 4 h. The formed formazan crystals were dissolved in 150 μL dimethyl sulfoxide (DMSO), and the absorbance was measured at 570 nm using a microplate reader (BioTek Synergy H1). Cell viability was calculated relative to untreated controls (n = 6), and the results are expressed as mean ± SD. Positive (10% DMSO) and negative (culture medium) controls were used for assay validation. Statistical analysis was performed using one-way ANOVA with Tukey’s post hoc test (p < 0.05, considered significant) [43–45].

### Surface wetting testing

The surface wettability of the hydrogels was evaluated by static contact angle measurements using a goniometer (DataPhysics OCA 15EC) equipped with a high-resolution CCD camera. Lyophilized hydrogel samples (10 × 10 × 2 mm³) were mounted on a stage and a deionized water droplet (5 µL) was gently dispensed onto the surface using a precision syringe. The contact angle was measured within 10 s of droplet deposition to minimize the evaporation effects. Five independent measurements were performed at different locations on each sample (n = 3 hydrogels per formulation) under ambient conditions (25°C, 60% RH). Data acquisition and analysis were performed using SCA20 software (DataPhysics), with results reported as the mean ± standard deviation. Hydrophilicity was further confirmed by measuring the water absorption capacity, where pre-weighed dry hydrogels were immersed in PBS (pH 7.4) until equilibrium swelling and the contact angle was reassessed on the swollen samples. Statistical significance was determined using the Student’s t-test (p < 0.05).

### Swelling study

The swelling properties of the hydrogels were evaluated using gravimetric analysis in different physiological buffers. Pre-weighed lyophilized hydrogel discs (10 mm diameter × 2 mm thickness, W_d_) were immersed in phosphate-buffered saline (PBS pH 7.4), acetate buffer (pH 5.0), and deionized water at 37°C under static conditions. At predetermined time intervals, samples were removed, blotted with filter paper to remove excess surface water, and weighed (W_t_). The swelling ratio (SR) was calculated as follows:


$$\text{SR}\;(\%)=[({\text{W}}_{\text{t}}-{\text{W}}_{\text{d}})/{\text{W}}_{\text{d}}]\times \text{100}$$ (1)

Swelling equilibrium was determined when three consecutive measurements showed <5% variation. All experiments were performed in triplicate (n = 3), and the data were analyzed using one-way ANOVA with post-hoc Tukey’s test (p < 0.05). The influence of BCDs incorporation on the swelling kinetics was assessed by comparison with control hydrogels without nanoparticles.

### Characterizations

The structural and physicochemical properties of the synthesized hydrogel nanocomposites were systematically characterized using advanced analytical techniques. Fourier-transform infrared spectroscopy (FTIR) analysis was conducted using a PerkinElmer Spectrum Two spectrometer in the range of 4000–400 cm ⁻ ¹ with 4 cm ⁻ ¹ resolution to identify functional groups and chemical interactions. Crystallinity was examined by X-ray diffraction (XRD) measurements on a Rigaku SmartLab diffractometer using Cu Kα radiation (λ = 1.5406 Å) at a scanning rate of 2° min ⁻ ¹ over the 2θ range of 5–80°. Optical properties were evaluated by photoluminescence (PL) spectroscopy using a Horiba Fluorolog-3 spectrofluorometer, and UV-Vis absorption spectra were recorded on a Shimadzu UV-2600 spectrophotometer in the 200–800 nm range. The photostability of the synthesized BCDs and BCD-incorporated hydrogel films was evaluated to assess their resistance to photobleaching under UV exposure. The samples were placed in a closed chamber and irradiated with a UV lamp (λ = 365 nm, 8 W) positioned at a fixed distance of 10 cm from the sample surface. Exposure was carried out at room temperature under ambient conditions for predetermined time intervals (0–120 min). After each interval, the fluorescence intensity of the samples was recorded using a fluorescence spectrophotometer to monitor changes in the emission intensity and peak position. Photostability was expressed as the relative fluorescence intensity (I/I₀), where I₀ and I correspond to the initial and post-irradiation intensities, respectively. Thermal stability was assessed by thermogravimetric analysis (TGA) on a TA Instruments Q50 analyzer under nitrogen atmosphere with a heating rate of 10°C min ⁻ ¹ from room temperature to 800°C. Morphological characterization was performed using field-emission scanning electron microscopy (FESEM; Carl Zeiss Sigma 300 VP) operated at 5 kV. The mechanical properties were evaluated through tensile and compression tests using an Instron 5966 universal testing machine equipped with a 500 N load cell at a crosshead speed of 5 mm/min.

### Cytotoxicity assessment (MTT assay)

The in vitro cytocompatibility of the developed hydrogel samples was evaluated using the MTT assay, following the principles outlined in ISO 10993–5:2009. Mouse fibroblast cells (L929, NCCS Pune, India) were used as the model cell line. The cells were cultured in Dulbecco’s modified Eagle’s medium (DMEM; high glucose; Gibco, USA) supplemented with 10% fetal bovine serum (FBS; Gibco, USA) and 1% penicillin-streptomycin (Thermo Fisher Scientific, USA) at 37 °C in a humidified atmosphere containing 5% CO₂. Hydrogel extracts were prepared by incubating sterilized hydrogel discs in fresh culture medium at a ratio of 0.2 g mL ⁻ ¹ for 24 h at 37 °C under gentle agitation. After incubation, the extracts were filtered through a 0.22 µm syringe filter to remove particulates before use. To ensure compliance with ISO 10993−5, three control groups were included. The blank control consisted of an extraction vehicle (DMEM) subjected to identical incubation conditions, without any hydrogel material. The negative control comprised cells cultured in fresh DMEM (stored at 4 °C prior to use) that were not been co-incubated with any test sample, representing the baseline cellular metabolic activity. The positive control consisted of cells treated with 0.1% (v/v) Triton X-100 to confirm the assay responsiveness. After 24 h exposure to the test extracts and controls, the medium was replaced with MTT solution (0.5 mg mL ⁻ ¹ in DMEM) and incubated for 4 h at 37 °C. The resulting formazan crystals were dissolved in DMSO, and the absorbance was recorded at 570 nm using a microplate reader (BioTek Synergy HTX, BioTek Instruments, Winooski, VT, USA). Cell viability (%) was calculated relative to that of the negative control group.

## Results and discussions

### Characterizations of BCDs

**Fig 2:**
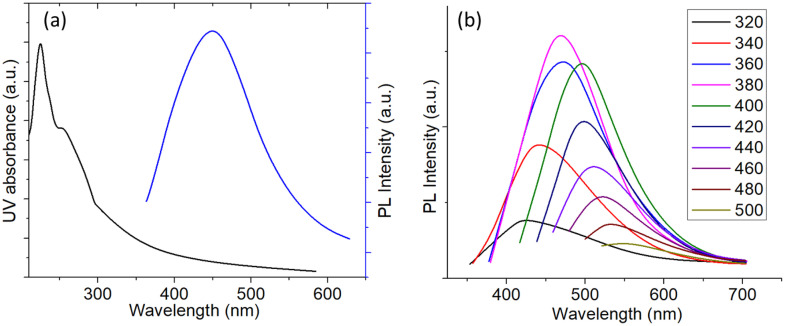
(a) UV and PL spectra of the BCDs in aqueous medium (b) excitation dependent PL spectra of the BCDs in aqueous medium.

The optical properties of the synthesized bagasse-derived carbon dots (BCDs) were systematically evaluated using UV–vis absorption and photoluminescence (PL) spectroscopy (Fig 2). The UV–vis spectrum of BCDs in aqueous dispersion (Fig 2a) exhibits a distinct absorption peak at approximately 275 nm, which is typically attributed to the π–π* transition of aromatic C = C bonds, indicating the presence of conjugated sp² carbon domains. A broad shoulder extending into the 300–350 nm region can be ascribed to the n–π* transitions of the C = O or C–O surface functionalities, suggesting abundant oxygen-containing groups that contribute to the hydrophilicity and surface reactivity of the BCDs. The PL spectrum recorded under 360 nm excitation (Fig 2a) shows a strong emission centered at approximately 450–460 nm, indicating the formation of blue-emitting carbon dots with high fluorescence intensity, which is consistent with surface defect–state mediated emission. The excitation-dependent PL behavior of the BCDs (Fig 2b) further confirms the heterogeneous distribution of emissive sites on the carbon dot surface. As the excitation wavelength increased from 320 to 500 nm, the emission maximum exhibited a red shift from ~440 to ~540 nm, accompanied by a gradual decrease in intensity. This phenomenon is characteristic of carbon-based nanomaterials with multiple emissive centers and varied particle sizes, where both quantum confinement effects and surface-state emissions contribute to the overall photoluminescence. The maximum fluorescence intensity was observed at an excitation wavelength of 360 nm, which correlated well with the absorption shoulder in the UV–vis spectrum, suggesting efficient excitation–emission coupling. These results collectively indicate that the hydrothermal synthesis of sugarcane bagasse biochar produced nanosized carbon dots with abundant surface functional groups, strong aqueous dispersibility, and tunable photoluminescence properties. The optical properties of the prepared hydrogels were examined under UV illumination (λ = 365 nm) to determine the photophysical influence of BCD incorporation. The pristine hydrogel film appeared optically transparent and exhibited no fluorescence, whereas the BCD-incorporated hydrogel showed bright blue emission. This distinct fluorescence confirms the successful integration of BCDs into the hydrogel matrix and originates from the surface states and π–π* transitions within the carbon dot structure. The presence of abundant functional groups in the hydrogel network likely enhances the stability and emission intensity of the embedded BCDs.

**Fig 3:**
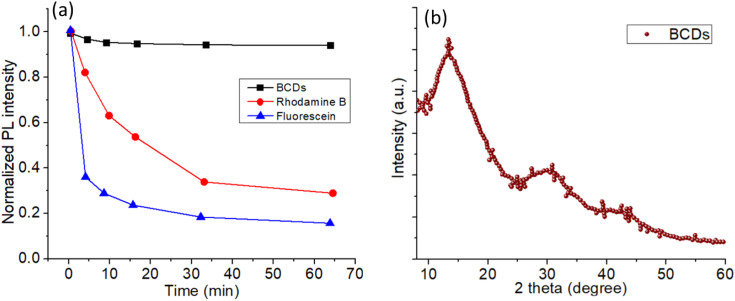
(a) Photostability analysis of the BCDs in comparison with rhodamine B and fluorescein (all in aqueous media) (b) XRD of the BCDs powder.

The photostability of the BCDs was evaluated in comparison with that of conventional organic fluorophores, rhodamine B and fluorescein, under continuous irradiation in an aqueous medium (Fig 3a). The BCDs exhibited exceptional stability, retaining more than 95% of their initial photoluminescence (PL) intensity even after 60 min of continuous light exposure. In contrast, rhodamine B and fluorescein undergo rapid photobleaching, and their PL intensities decrease to approximately 30% and 15% of the initial values, respectively, within the same duration [46]. The superior photostability of BCDs can be attributed to their robust sp² carbon core and stable surface passivation, which prevents photo-induced degradation and quenching. This stability is highly advantageous for applications in long-term bioimaging and optical sensing, where sustained fluorescence is critical. The structural characteristics of the BCDs were examined using X-ray diffraction (XRD) analysis (Fig 3b). The XRD pattern displayed a broad diffraction peak centered at approximately 2θ ≈ 22°, which is characteristic of amorphous carbon materials with a predominantly turbostratic structure [47]. The absence of sharp crystalline peaks indicates the lack of long-range order, which is consistent with the nanoscale dimensions and abundant surface functionalization of the carbon dots [48]. The broad diffraction profile further suggests the presence of disordered graphitic domains interspersed with oxygen-containing groups, corroborating the UV–vis observations of the π–π* and n–π* transitions.

**Fig 4:**
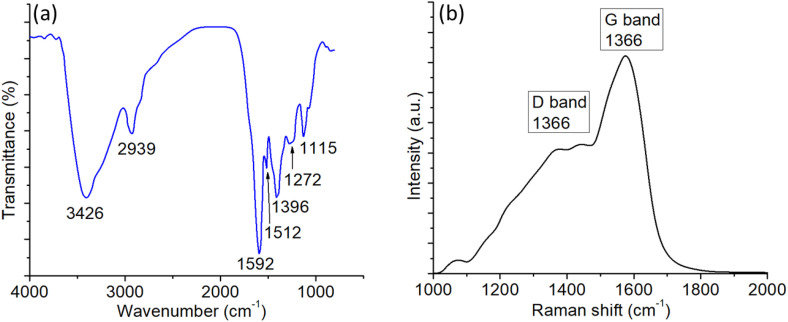
(a) FTIR spectrum of BCDs powder (after lyophilization) (b) Raman spectrum of the BCDs powder.

The chemical bonding and structural characteristics of the BCDs were further examined using Fourier-transform infrared (FTIR) and Raman spectroscopy (Fig 4). The FTIR spectrum of the lyophilized BCD powder (Fig 4a) exhibited a broad absorption band centered around 3400 cm ⁻ ¹, corresponding to O–H and N–H stretching vibrations, indicative of the surface hydroxyl and amino groups. The peaks observed at ~2920 and 2850 cm ⁻ ¹ were assigned to the C–H stretching vibrations of aliphatic chains. A strong peak near 1720 cm ⁻ ¹ corresponded to C = O stretching of carbonyl or carboxyl groups, while the band at ~1620 cm ⁻ ¹ was attributed to C = C stretching in aromatic domains or C = O bending of amide functionalities [49]. Additional peaks in the 1000–1200 cm ⁻ ¹ region were assigned to the C–O–C and C–O stretching vibrations, confirming the presence of oxygen-rich functional groups. These observations indicate that the BCD surface contains abundant polar functionalities, which enhance the aqueous dispersibility and provide reactive sites for further modification. The Raman spectrum of the BCD powder (Fig 4b) shows two prominent peaks: the D band at ~1350 cm ⁻ ¹, corresponding to disordered sp³ carbon and structural defects, and the G band at ~1580 cm ⁻ ¹, associated with the E₂g mode of sp²-hybridized graphitic carbon. The intensity ratio (I_D_/I_G_) was greater than 1, suggesting a high degree of disorder and small graphitic domain size, consistent with the amorphous nature observed in the XRD pattern (Fig 3b) [50]. The presence of both the D and G bands confirms that the BCDs consist of graphitic cores interspersed with defect sites, which likely contribute to the excitation-dependent photoluminescence behavior discussed earlier.

### Synthesis and characterizations of BCDs based nanocomposite hydrogels

**Fig 5:**
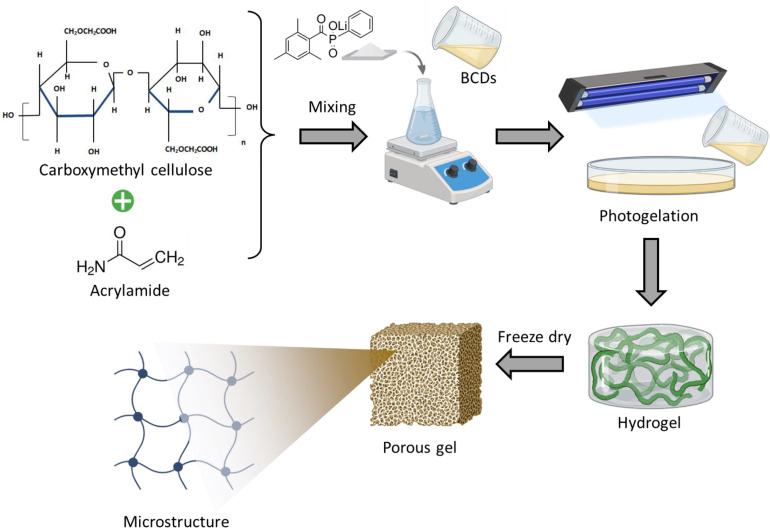
Schematic of BCDs loaded *in situ* nanocomposite hydrogel synthesis.

The formation of the BCD-loaded in situ nanocomposite hydrogel was confirmed through the schematic representation shown in Fig 5, which illustrates the integration of carboxymethyl cellulose (CMC), acrylamide (AM), and photopolymerization in the presence of BCDs. The process begins with the homogeneous mixing of CMC and AM, followed by the incorporation of BCDs and a photoinitiator, enabling the uniform dispersion of the nanofillers within the precursor matrix. Upon UV irradiation, rapid photogelation occurs, leading to the formation of a three-dimensional crosslinked polymeric network in which the BCDs are embedded at the nanoscale. The freeze-dried hydrogel exhibited a highly porous interconnected microstructure, as depicted in Fig 5. This morphology is favorable for high water absorption, efficient nutrient/drug diffusion, and improved mechanical integrity. The homogeneous distribution of BCDs within the polymer network is expected to enhance the mechanical strength of the hydrogel through nanofiller–polymer interactions while simultaneously imparting stable fluorescence for real-time monitoring and potential bioimaging applications. Moreover, the abundant functional groups on both CMC and BCDs provide reactive sites for hydrogen bonding and potential covalent interactions, contributing to the network stability and stimuli-responsive behavior.

**Fig 6:**
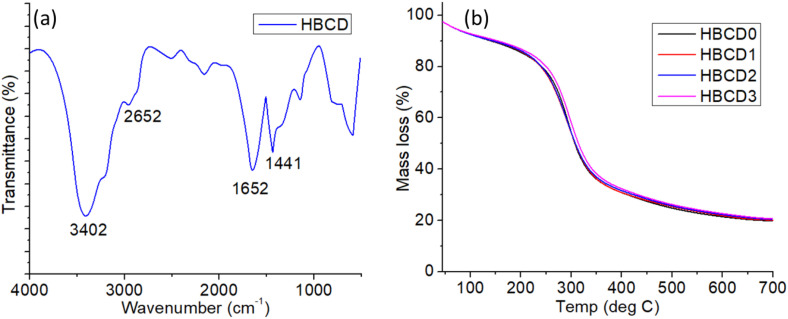
(a) FTIR spectrum of BCD loaded nanocomposite hydrogel (b) TGA plot of the pure hydrogel (HBCD0) and BCDs loaded nanocomposite hydrogels.

The chemical structure and thermal stability of the BCD-loaded nanocomposite hydrogels were evaluated by FTIR and TGA (Fig 6). The FTIR spectrum (Fig 6a) of the hydrogel containing BCDs (HBCD) displayed a broad absorption band at 3402 cm ⁻ ¹, corresponding to O–H and N–H stretching vibrations, confirming the presence of hydroxyl and amide groups on the CMC, AM, and BCD surface functionalities. The peak at 2652 cm ⁻ ¹ can be attributed to C-H stretching, while the band at 1652 cm ⁻ ¹ corresponds to the C = O stretching of amide I and/or carboxyl groups. The absorption at 1441 cm ⁻ ¹ is associated with C-N stretching or CH₂ bending vibrations [51]. Additional peaks in the fingerprint region (<1200 cm ⁻ ¹) are related to C-O-C and C-O stretching, confirming the incorporation of oxygen-rich groups from both the polymeric matrix and BCDs. These spectral features collectively indicate the successful integration of BCDs into the hydrogel network through both physical entrapment and potential hydrogen-bonding interactions. The thermal degradation behaviors of the pure hydrogel (HBCD0) and BCD-loaded hydrogels (HBCD1-HBCD3) were investigated using TGA (Fig 6b). All the samples exhibited a similar two-step degradation profile. The initial weight loss below ~150 °C corresponds to the evaporation of bound and adsorbed water. The major degradation step, occurring between 250–400 °C, was attributed to the decomposition of the CMC/AM polymer backbone and partial breakdown of the BCD surface functionalities. A residual mass above 500 °C reflects a thermally stable carbonaceous structure [52]. Notably, the presence of BCDs did not significantly alter the overall degradation temperature profile, suggesting that nanofiller incorporation maintained the intrinsic thermal stability of the hydrogel while imparting additional functional properties [53]. These results confirm that BCDs are effectively embedded within the hydrogel matrix without compromising their thermal robustness, making the material suitable for applications that require stability under physiological and moderately elevated temperatures.

**Fig 7:**
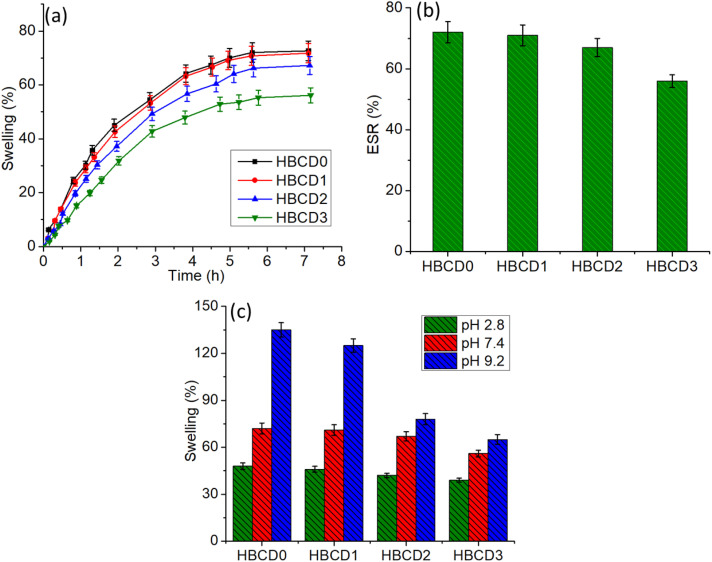
(a) Swelling kinetics study of the unloaded (with BCDs) and with BCDs (nanocomposite) hydrogels (b) Equilibrium swelling behavior of the hydrogels (c) Swelling behavior of the hydrogels are different pH environments.

The swelling performances of the pure hydrogel (HBCD0) and BCD-loaded nanocomposite hydrogels (HBCD1–HBCD3) were systematically evaluated under different conditions (Fig 7). The swelling kinetics (Fig 7a) revealed that all the hydrogels exhibited a rapid increase in water uptake during the first 2 h, followed by a gradual approach to equilibrium. HBCD0 showed the highest equilibrium swelling ratio, while increasing the BCD content led to a progressive decrease in the swelling capacity, with HBCD3 exhibiting the lowest value. This reduction can be attributed to denser crosslinking and enhanced filler–polymer interactions in the presence of higher BCD concentrations, which restrict polymer chain mobility and limit network expansion [54]. The equilibrium swelling ratio (ESR) values (Fig 7b) further confirmed this trend, with HBCD0 and HBCD1 exhibiting ESRs above 65%, whereas HBCD2 and HBCD3 showed noticeably lower values. The decrease in the ESR with increasing BCD content is consistent with the hypothesis that BCD incorporation reinforces the hydrogel network, thereby reducing the free volume available for water penetration. The pH-responsive swelling behavior (Fig 7c) demonstrated that all hydrogel formulations were sensitive to the environmental pH [55]. Swelling was minimal at acidic pH (2.8), increased at physiological pH (7.4), and reached maximum values under alkaline conditions (pH 9.2). This pH-dependent swelling can be attributed to the ionization of carboxyl and hydroxyl groups within the CMC backbone, which enhances electrostatic repulsion and water uptake at higher pH. Notably, even under alkaline conditions, the swelling capacity decreased with higher BCD loading, indicating that the reinforcing effect of BCDs dominates pH-induced expansion.

### Mechanical properties

**Fig 8:**
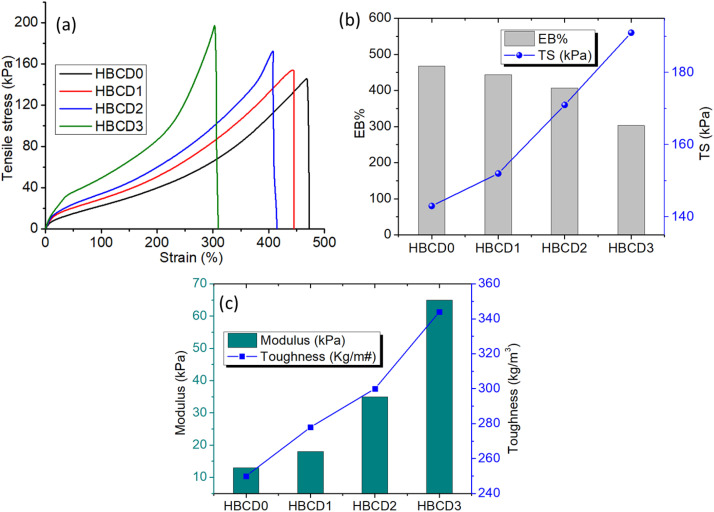
(a) Tensile behavior of the nanocomposite hydrogels against uniaxial tension mode (b) Elongation at break (EB%) and Tensile strength (TS) histogram plot of the hydrogels (c) Elastic modulus and toughness of the hydrogels.

The mechanical performance of the pure hydrogel (HBCD0) and BCD-loaded nanocomposite hydrogels (HBCD1–HBCD3) was evaluated under uniaxial tensile testing, and the results are shown in Fig 8. The tensile stress–strain curves (Fig 8a) demonstrate a significant improvement in the tensile strength and elongation with increasing BCD content. While HBCD0 exhibited the lowest tensile stress and moderate elongation, the incorporation of BCDs progressively enhanced both properties, with HBCD3 exhibiting the highest tensile strength and strain at break. This improvement can be attributed to the strong interfacial interactions between the BCDs and polymeric matrix, which facilitated effective stress transfer and restricted polymer chain slippage. The elongation at break (EB%) and tensile strength (TS) values (Fig 8b) further illustrate this trend. EB% increased notably from HBCD0 to HBCD3, indicating improved elasticity, while TS also increased steadily, suggesting that the reinforcing effect of BCDs not only maintains but also enhances the structural integrity of the hydrogel under deformation. Such enhancement is likely due to a combination of physical entanglement and hydrogen bonding between the hydroxyl/carboxyl groups of the BCDs and the hydrogel network [56]. The elastic modulus and toughness values (Fig 8c) showed a consistent increase with higher BCD loading. The modulus increased from ~7 kPa for HBCD0 to over 60 kPa for HBCD3, indicating a substantial improvement in stiffness. Likewise, toughness, which represents the energy absorbed before fracture, increased from ~245 kg/m³ for HBCD0 to ~350 kg/m³ for HBCD3, confirming that BCD incorporation enhances both the strength and energy dissipation capacity.

### Morphology and surface properties

**Fig 9:**
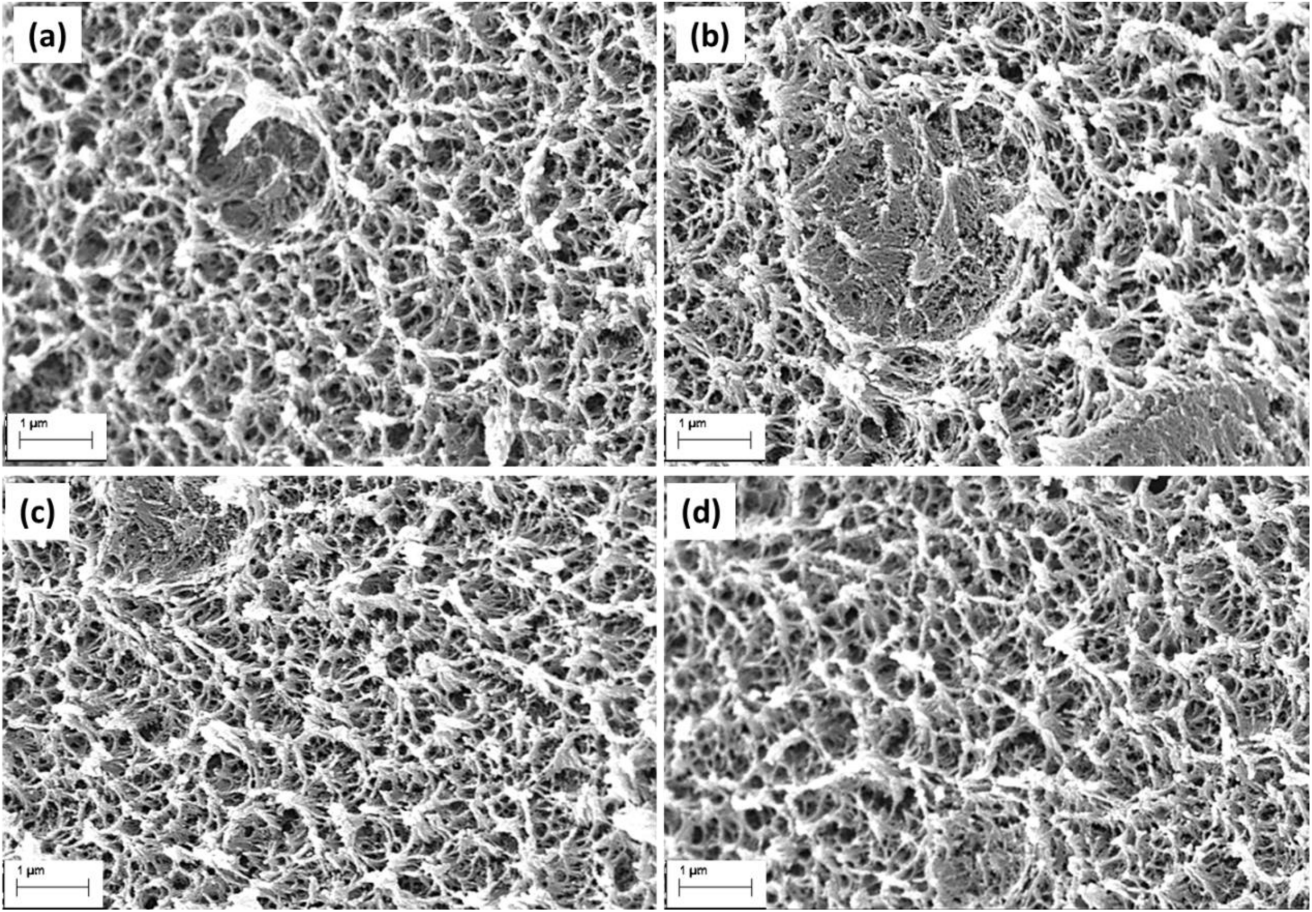
SEM images of the (a) HBCD0 (b) HBCD1 (c) HBCD2 and (d) HBCD3 hydrogels showing their porous morphologies.

**Fig 10:**
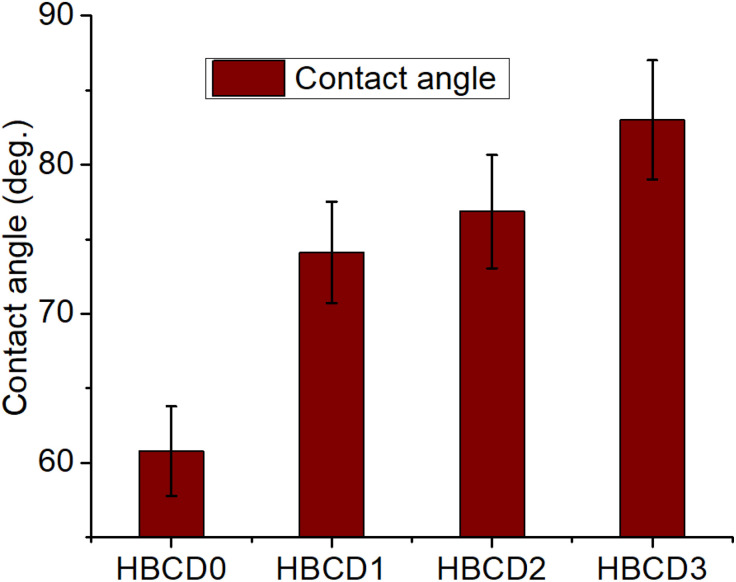
Surface wettability (contact angle) measurements of the nanocomposite hydrogels.

The microstructural features of the unmodified hydrogel (HBCD0) and nanocomposite hydrogels containing varying concentrations of boron-doped carbon dots (HBCD1–HBCD3) were investigated by SEM, as shown in Fig 9(a-d). All the samples exhibited a three-dimensional interconnected porous architecture, which is a hallmark of physically crosslinked hydrogel networks. In HBCD0, the pores were relatively uniform with smooth internal walls, indicating a well-organized polymer chain arrangement during gelation. The introduction of BCDs into the hydrogel matrix (HBCD1–HBCD3) resulted in noticeable morphological changes. Specifically, the pore walls became rougher and denser, accompanied by the formation of irregular voids and microprotrusions. This structural transformation can be attributed to strong hydrogen bonding and electrostatic interactions between the functional groups of the polymer backbone and the oxygen-/boron-containing moieties on the BCD surfaces. Such interactions are likely to enhance the local crosslinking density, leading to a tighter and more robust network [57]. For HBCD1 and HBCD2, the pores appeared to be moderately smaller and more irregular compared to HBCD0, suggesting a partial restriction of the polymer chain mobility during network formation. In HBCD3, the pore structure was the most compact, with pronounced surface corrugations and thicker pore walls, indicating a significant reinforcement effect from high BCD loading. The increased network density in HBCD3 enhanced the mechanical strength and reduced the pore collapse under deformation, which correlates with the mechanical performance trends observed in Fig 8. Furthermore, the homogeneous distribution of BCDs throughout the matrix implies effective dispersion and strong interfacial compatibility, which are critical for maintaining the structural integrity during swelling–deswelling cycles. The wettability properties of the hydrogels were assessed using static contact angle measurements (Fig 10). HBCD0 exhibited a low contact angle (~60°), which is characteristic of a highly hydrophilic material with strong water–polymer interactions. Hydrophilicity arises from abundant polar functional groups, such as hydroxyl and amide moieties in the polymer chains, facilitating rapid water spreading and absorption [58]. Upon incorporation of BCDs, a progressive increase in the contact angle was observed: ~ 72° for HBCD1, ~ 76° for HBCD2, and ~85° for HBCD3. This trend indicates a gradual shift towards a more hydrophobic surface with increasing BCD content. This change can be explained by two main factors: (i) partial shielding of hydrophilic groups on the polymer backbone due to BCD–polymer interactions and (ii) the presence of less polar aromatic and boron-containing domains on the BCD surfaces, which lowers the surface free energy. This increase in hydrophobicity may slow the initial rate of water uptake, which could be advantageous for applications that require controlled swelling and prolonged mechanical stability in aqueous environments. The observed correlation between the surface morphology (denser pore network) and wettability suggests that microstructural compaction also contributes to reduced surface water affinity.

### Drug release study in *in vitro* model

**Fig 11:**
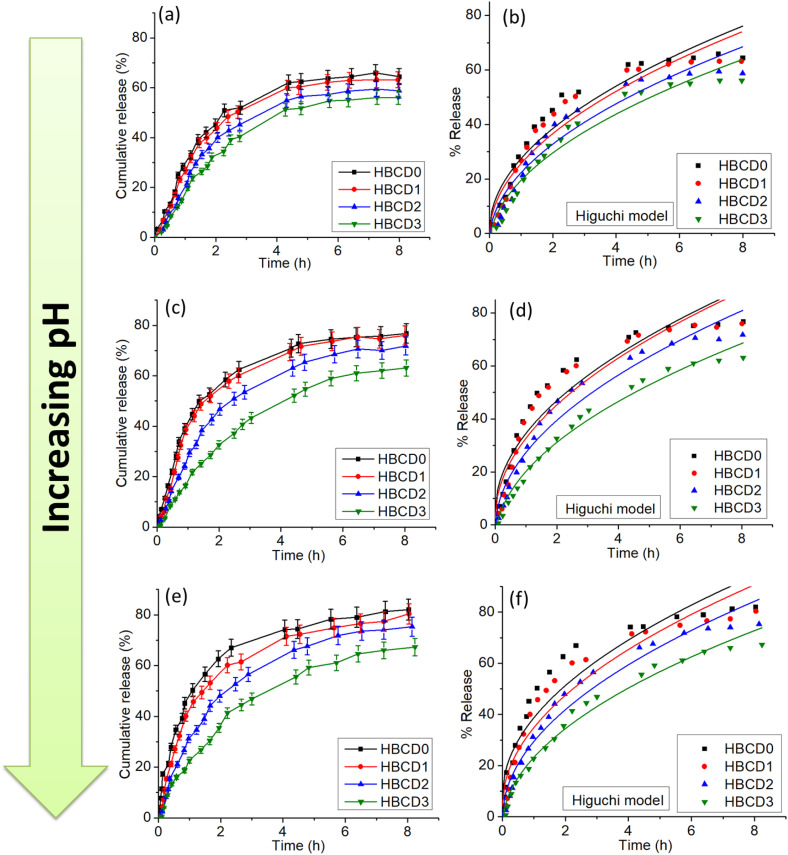
Cumulative release behavior of the hydrogels as a function of pH. (a) Cumulative release profile of the hydrogels at pH 3.8 (b) Higuchi model fittings plot at pH 3.8 (c) Cumulative release profile of the hydrogels at pH 7.4 (d) Higuchi model fittings plot at pH 7.4 (e) Cumulative release profile of the hydrogels at pH 9.2 (d) Higuchi model fittings plot at pH 9.2.


The pH-responsive drug-release behavior of the hydrogels (HBCD0–HBCD3) was evaluated at pH 3.8, 7.4, and 9.2 to simulate acidic, physiological, and mildly alkaline environments, respectively. Figs 11a, 11c, and 11e show the cumulative release profiles, while Figs 11b, 11d, and 11f show the corresponding fits to the Higuchi model. The Higuchi model is a widely used mathematical framework for describing drug release from planar matrices, particularly when release is controlled by diffusion through a hydrated polymer network [59]. The governing equation is as follows:

$$Q=k_{H}\sqrt{t}$$

Where Q is the amount of drug released at time t and k_H_ is the Higuchi release constant), assumes that drug diffusion is the primary transport mechanism, the initial drug concentration is much higher than the solubility limit, and matrix swelling or erosion is minimal during the early stages of release.

In this study, the excellent linear fits across all pH conditions demonstrated that the release of the encapsulated drug from HBCD hydrogels is primarily governed by Fickian diffusion through the polymer network, with swelling effects playing a secondary role. The ability to fit the data to the Higuchi model across a range of pH values confirms the robustness of the release mechanism and provides a quantitative way to compare the release rates (k_H_) between formulations and environments [60]. At pH 3.8 (Fig 11a), all hydrogels showed a rapid initial burst release during the first 2 h, followed by a slower, sustained release up to 8 h. The control hydrogel (HBCD0) reached ~78% cumulative release, whereas BCD-containing hydrogels showed reduced release rates, with HBCD3 releasing ~68%. This decrease in release with higher BCD content is attributed to a denser polymeric network formed through hydrogen bonding and electrostatic interactions between the BCD functional groups and polymer chains. Under acidic conditions, protonation of ionizable groups enhances these interactions, reducing hydrogel swelling and hindering drug diffusion [61]. The Higuchi plots (Fig 11b) displayed strong linearity (R² > 0.97), confirming that the release mechanism was predominantly Fickian diffusion, which is the theoretical basis of the Higuchi model. At pH 7.4 (Fig 11c), the release rates increased compared to pH 3.8, with cumulative release reaching ~83% for HBCD0 and ~74% for HBCD3. The neutral environment leads to partial deprotonation of the acidic groups, reducing the electrostatic attraction between the polymer chains and BCDs, resulting in greater swelling and enhanced diffusivity. The corresponding Higuchi plots (Fig 11d) showed high correlation coefficients (R² > 0.96), indicating that diffusion remained the dominant mechanism even under physiological swelling conditions. At pH 9.2 (Fig 11e), all hydrogels exhibited the highest release rates, exceeding 85% for HBCD0 and 79% for HBCD3 after 8 hours. Under alkaline conditions, full deprotonation of the acidic moieties generates strong electrostatic repulsion, causing extensive network expansion and promoting drug mobility. Differences between BCD-containing and control hydrogels were minimal, as high swelling minimized the restrictive effect of the network [62]. The Higuchi fitting at pH 9.2 (Fig 11f) again showed excellent linearity (R² > 0.95). Although the experimental release points show slight visual deviations from the ideal linear trend, the Higuchi model provided a statistically reliable fit for our system. The linear regression yielded an R² value greater than 0.95, meeting the 95% confidence requirement. Minor deviations are expected because the Higuchi model assumes purely diffusion-controlled release and constant matrix geometry, while the actual system may undergo slight structural relaxation or variations in the diffusion pathway. Despite these effects, the high R² value indicates that diffusion remains the dominant mechanism, supporting the use of the Higuchi model for describing the release behavior.

### Cytotoxicity analysis

**Fig 12:**
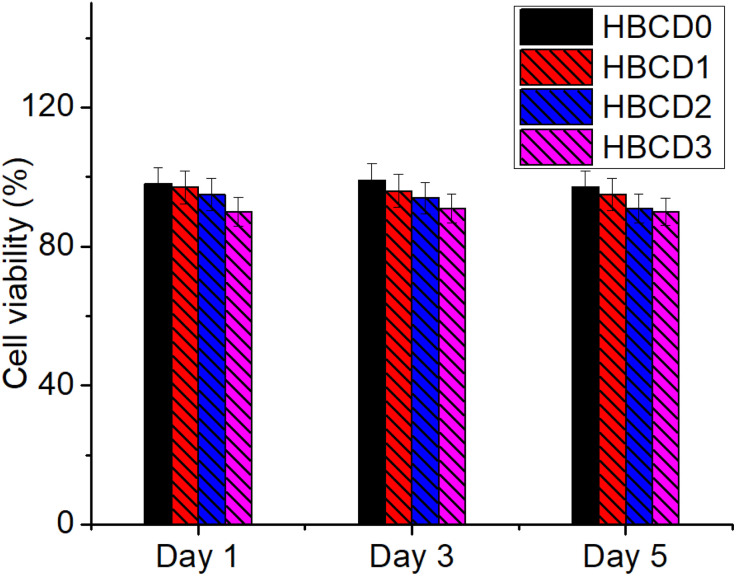
Cytotoxicity evaluation of the BCDs free and other nanocomposites hydrogels against live cells after 1^st^, 3^rd^, and 5^th^ day. Data were presented as mean ± standard deviation, n = 3.

The biocompatibility of the prepared hydrogels was assessed by evaluating their cytotoxicity toward live cells over a 5-day incubation period. Fig 12 shows the cell viability results for the BCD-free control hydrogel (HBCD0) and BCD-containing nanocomposite hydrogels (HBCD1–HBCD3) on days 1, 3, and 5, with data expressed as mean ± standard deviation (n = 3). Figure S1 in S1 File. shows cell proliferation microscopy images of live fibroblasts for the HBCD3 sample after the 3rd and 5th day of culture, providing visual evidence of cell growth and viability.

On day 1, all hydrogel formulations maintained high cell viability (> 85%), indicating minimal acute cytotoxic effects immediately after exposure [63]. The control hydrogel (HBCD0) and low-BCD-content hydrogel (HBCD1) showed slightly higher viability than the higher-BCD-content samples (HBCD2 and HBCD3), which may be attributed to the initial ion release or residual surface groups from the BCDs interacting with cell membranes. However, these differences were not statistically significant (P > 0.05). By day 3, the viability of all samples remained above 80%, with a slight upward trend compared to day 1, suggesting that any initial cellular stress was transient and that the cells adapted to the hydrogel environment. This improvement may be related to the reduced leaching of unbound nanomaterials over time and to the development of a protein corona on the hydrogel surface in the culture medium, mitigating direct nanoparticle–cell contact. On day 5, cell viability values were maintained or further improved, with most samples exceeding 85–90% [64]. This sustained high viability confirmed that BCD-containing hydrogels did not induce chronic cytotoxic effects under the tested conditions. Notably, the slight difference in viability between the BCD-rich and BCD-free hydrogels observed on day 1 was no longer apparent, indicating that the polymeric network effectively stabilized the embedded nanocomposites over time.

## Conclusions

In this study, we successfully developed biocompatible nanocomposite hydrogels incorporating heteroatom-doped carbon dots (BCDs) derived from sugarcane bagasse and systematically evaluated their drug release behavior under physiologically relevant pH conditions as well as their cytotoxicity toward live cells. The incorporation of BCDs into the hydrogel network significantly enhanced its structural and functional properties, including its mechanical stability, fluorescence, and tunable swelling behavior. The pH-responsive release profiles demonstrated that ibuprofen release was faster under acidic conditions and slower at higher pH values, highlighting the capacity of the hydrogel to modulate drug release according to the local chemical environment. This behavior is particularly relevant for targeted drug delivery applications where localized pH variations in tissues or biological compartments can be leveraged to achieve controlled release. Analysis using the Higuchi diffusion model indicated that the release kinetics were predominantly diffusion-controlled, emphasizing the role of both the hydrogel matrix and the embedded BCDs in regulating mass transport. The BCDs contribute not only to the mechanical reinforcement of the hydrogel but also to the formation of microdomains that facilitate controlled diffusion of the drug molecules. The pH-responsive swelling of the hydrogel network, combined with the intrinsic diffusion behavior of ibuprofen within the BCD-reinforced matrix, provided a clear mechanism for the observed release profiles. Cytotoxicity assays over a five-day period confirmed sustained high cell viability (>80–90%), demonstrating the biocompatibility and cytosafety of the developed hydrogels. The observed fluorescence of the BCDs within the hydrogel further allowed for the potential monitoring of drug-loaded matrices in situ, adding an additional functional advantage for biomedical applications. Taken together, these findings establish the developed BCD-based nanocomposite hydrogel as a versatile and robust platform for controlled, pH-responsive drug delivery. The system offers tunable release rates dictated by the environmental pH and hydrogel structure, while demonstrating promising cytocompatibility and potential suitability for biomedical applications, contingent upon further biological applications. This study lays a strong foundation for further optimization of hydrogel formulations for therapeutic use, highlighting their potential for practical applications in drug delivery systems where site-specific, controlled release is desired.

## Supporting information

S1 File The data supporting the findings of this study are provided in the Supporting Information file intended for publication. (DOCX)
