# Supplementary material for: Bio-waste derived fluorescent carbon dots based biodegradable mechanically strong hydrogel for stimuli-responsive nonsteroidal anti-inflammatory drug (NSAID) delivery
Source: PLoS One. 2026 Jan 23;21(1):e0340974. doi: 10.1371/journal.pone.0340974 (PMC12829963; doi:10.1371/journal.pone.0340974)
Supplement: S1 File — (DOCX) [file pone.0340974.s001.docx]

Supporting information of

**Bio-Waste Derived Fluorescent Carbon Dots Based Biodegradable Mechanically Strong Hydrogel for Stimuli-Responsive Nonsteroidal Anti-Inflammatory Drug (NSAID) Delivery**

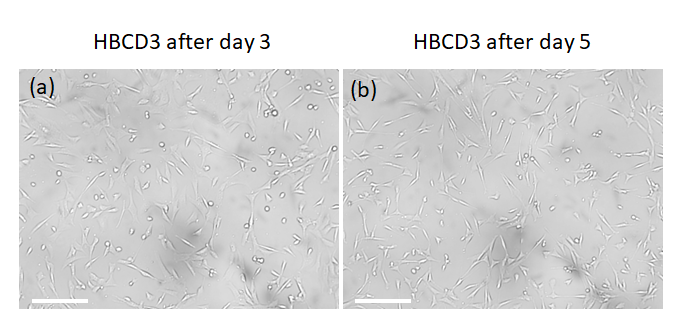


**Figure S1.** Cell proliferation microscopy images of live fibroblasts for HBCD3 sample after 3^rd^ and 5^th^ day of culture.
